# Serum Thiols and Ischemia-Modified Albumin Levels in Children With Major Depressive Disorder: A Cross-Sectional Study

**DOI:** 10.7759/cureus.65861

**Published:** 2024-07-31

**Authors:** Merve Okuyucu, Mehmet Fatih Ceylan, Selma Tural Hesapcioglu, Fatma Sena Konyalioglu, Esra Firat Oguz, Salim Neselioglu

**Affiliations:** 1 Department of Child and Adolescent Psychiatry, Ministry of Health Dogubayazit Dr. Yasar Eryilmaz State Hospital, Agri, TUR; 2 Department of Child and Adolescent Psychiatry, Ankara Yıldırım Beyazıt University Faculty of Medicine, Ankara, TUR; 3 Department of Public Health, Adıyaman Provincial Health Directorate, Adiyaman, TUR; 4 Department of Biochemistry, Ankara Yıldırım Beyazıt University Faculty of Medicine, Ankara, TUR

**Keywords:** oxidative stres, thiol, biomarkers, adolescent, depression

## Abstract

Introduction

This cross-sectional study aimed to investigate potential biomarkers of oxidative stress by analyzing serum thiol-disulfide homeostasis (TDH) and ischemia-modified albumin (IMA) levels in children who have been diagnosed with major depressive disorder (MDD).

Methods

The study included 24 medication-naive children aged seven to 17 years diagnosed with MDD and 19 healthy controls matched for age, gender, and body mass index. Clinical interviews were conducted using the Schedule for Affective Disorders and Schizophrenia for School-Age Children-Present and Lifetime Version (K-SADS-PL-DSM-5), and diagnoses were made according to DSM-5. The Child Depression Inventory (CDI) and the Clinical Global Impression Scale-Severity (CGI-S) were also administered to the participants. Venous blood samples were taken from all subjects to assess TDH and IMA levels, considered potential oxidative stress indicators.

Results

The study showed no significant difference in TDH and IMA levels between the MDD and the control groups. Although not statistically significant, it was observed that native thiol and total thiol levels were higher in the MDD group. No direct relationship was found between disease severity and either TDH or IMA levels.

Conclusion

In conclusion, while there were no significant differences in the levels of TDH and IMA, higher levels of both native and total thiols were found in the MDD group.

## Introduction

Major depressive disorder (MDD) is one of the most prevalent psychiatric disorders affecting children and adolescents (hereafter it is referred to as children). Childhood prevalence rates of MDD range from 1.3% to 8%, with a lifetime prevalence of about 19% [1,2]. It has many negative consequences, such as school failure, school dropout, and difficulty finding future employment [3], while also significantly increasing the risk of suicide [4]. Given these serious consequences, the increasing prevalence in children over the past decade [2] has led to a growing interest in understanding how MDD develops [5].

Oxidative stress, characterized by an imbalance between oxidants and antioxidants, has emerged as a notable candidate in research on the etiology of MDD. This imbalance culminates in detrimental effects on cellular components, potentially inducing apoptosis and cell death [6]. The brain, characterized by elevated oxygen consumption and a lipid-rich environment, is particularly susceptible to oxidative stress, thereby suggesting its involvement in diverse psychiatric disorders [7]. In the pathophysiology of MDD, oxidative stress plays an important role [8]. There is a link between increased oxidative stress and adverse outcomes associated with depression [9], highlighting the complex effects of oxidative stress on vital biomolecules and neuronal function in affected individuals [8]. Despite the recognition of oxidative stress as a possible factor in the etiology of MDD, understanding the specific biochemical mechanisms involved have remained crucial. Thiol-disulfide homeostasis (TDH) and ischemia-modified albumin (IMA) have been proposed as potential indicators of oxidative imbalance in biological systems [10,11].

Thiols act as antioxidants and are represented by a sulfhydryl group (-SH). They can undergo oxidation reactions, forming reversible disulfide bonds (SS). These bonds act as protective mechanisms against damage caused by reactive oxygen species in tissues and cells. The balance between oxidants and antioxidants, known as TDH, is crucial as it plays a role in various biochemical processes such as regulating protein function, influencing enzyme function, and controlling transcription. It can be measured by the ratio of reduced thiols to disulfide bonds [12]. The role of TDH has been studied in many psychiatric disorders in adults. However, fewer studies have examined the relationship between TDH and MDD, and those that have reported inconsistent results [10]. IMA is a new marker of oxidative stress. It is produced as a result of albumin damage under hypoxic conditions [11]. Cross-sectional studies of adults with MDD [13] and other depressive disorders [14] have yielded conflicting findings.

A limited number of studies have examined the relationship between MDD and oxidative stress using various biomarkers and have found increased oxidative stress in childhood [15-17]. While one recent study has evaluated the association between TDH and MDD [18], there is a lack of research using IMA in this context. To date, there has been no specific investigation of the relationship between MDD and oxidative stress in childhood using both TDH and IMA.

Environmental and biological factors [9] and their interactions have been implicated in the etiology of MDD. However, identifying the causes of MDD in children remains a major challenge. Importantly, this cross-sectional study represents one of the first attempts to investigate oxidative stress in children with MDD by assessing both TDH and IMA levels. We hypothesized that there would be a deterioration in TDH and an increase in IMA levels.

## Materials and methods

Participants and procedure

Twenty-four children diagnosed with MDD, ranging in age from seven to 17 years, were recruited based on meeting the diagnostic criteria outlined in the Diagnostic and Statistical Manual of Mental Disorders, 5th edition (DSM-5) [19]. Nineteen age- and gender-matched healthy control (HC) subjects were used as the control group. All subjects were recruited from the Child and Adolescent Psychiatry Outpatient Clinic of Ankara Yıldırım Beyazıt University Yenimahalle Training and Research Hospital.

The assessments were conducted by child and adolescent psychiatrists under the supervision of experienced child and adolescent psychiatrists and/or professors. Patients who met the diagnostic criteria for MDD according to the DSM-5 were included in the case group. The inclusion criteria for the case group were as follows: first-time diagnosis of MDD; no psychiatric diagnoses other than MDD; and no psychotropic treatment before study entry. The HC group comprised children who attended the outpatient clinic and did not receive a psychiatric diagnosis. The exclusion criteria for both groups were as follows: age seven years or younger and 17 years or older; chronic diseases, including metabolic, genetic, endocrine, neurological, and blood disorder-like conditions such as anemia; below-average intelligence; past or current use of psychotropic medications; smoking, alcohol, or drug use; recent infections within the past month; and body mass index (BMI) outside the normal range for age [20].

All participants were assessed for psychiatric disorders using the Schedule for Affective Disorders and Schizophrenia for School-Age Children-Present and Lifetime Version, based on DSM-5 criteria (K-SADS-PL-DSM-5) [21]. The researchers administered the Clinical Global Impression Scale-Severity (CGI-S) to assess symptom severity [22]. In addition, all subjects completed the Child Depression Inventory (CDI) [23]. Height and weight were then measured by nurses, and BMI was calculated from these measurements.

Venous blood samples (10 mL per patient) were collected from the antecubital vein into biochemistry tubes at 9:00 am after a 12-hour overnight fast. Overnight fasting was recommended, meaning no food or drink after 9:00 pm the night before the blood test, due to the potential increase in oxidative stress caused by elevated blood glucose levels [24]. The samples were incubated for 30 minutes and then centrifuged at 2000×g for 15 minutes. The resulting serum samples were stored at -80°C for subsequent analysis of serum IMA levels and TDH activity.

The parameters of TDH were evaluated using an automated spectrophotometric method developed by Erel and Neselioglu (2014) [12]. The disulfide bonds were first reduced to free functional thiol groups using sodium borohydride (NaHB_4_). To eliminate any residual NaHB_4_, formaldehyde was used to prevent interference with the reducing agent 5,5′-dithiobis-(2-nitrobenzoic) acid (DTNB). All thiol groups, including both the reduced and the original thiol groups, were then quantified after reaction with DTNB. From these measurements, the study derived values for native (SH) and total thiols (SH + SS), disulfide amounts (SS), disulfide to total thiol percentage ratios (SS/SH + SS), disulfide to native thiol percentage ratios (SS/SH), and native thiol to total thiol percentage ratios (SH/SH + SS). In addition, the dynamic disulfide amount was calculated as half the difference between total and native thiols.

Serum levels of IMA were assessed using the albumin cobalt binding assay, a spectrophotometric method developed by Bar-Or and colleagues (2000) to measure the cobalt binding capacity of albumin, expressed in absorbance units (ABSU) [25].

The study was conducted as a cross-sectional study from October 15, 2022, to January 15, 2023, at the outpatient department of the Child and Adolescent Psychiatry Clinic of Ankara Yıldırım Beyazıt University Yenimahalle Training and Research Hospital, Ankara, Turkey. Approval was obtained from the Ethics Committee of Ankara Yildirim Beyazit University Yenimahalle Education and Research Hospital (protocol number: E-2022-59, approval dated: 09/28/2022). The study adhered to the principles of the Declaration of Helsinki as revised in 2000, ensuring the welfare and rights of the participants. Verbal consent was obtained from all participants, and written informed consent was obtained from their legal guardians.

Measurements

Schedule for Affective Disorders and Schizophrenia for School-Age Children-Present and Lifetime Version (K-SADS-PL-DSM-5)

The K-SADS-PL-DSM-5 is a semi-structured diagnostic interview revised by Kaufman et al. (2016) to screen for psychopathology in children aged six to 18 years, according to DSM-5 diagnostic criteria. Certified child psychiatrists conduct the interview, taking into account responses from both parents/caregivers and children to ensure a thorough assessment of each symptom across different psychiatric disorders in children [21]. A Turkish adaptation of this scale was conducted, and its validity was assessed by Unal et al. (2019) [26].

Child Depression Inventory (CDI)

CDI is a 27-item self-rating scale developed to assess depressive symptoms in children over the past two weeks. Each item is scored on a three-point scale reflecting the severity of symptoms (zero for none, one for moderate, and two for severe) [23]. Oy (1991) conducted a Turkish validation and reliability study of this scale, recommending a cut-off score of 19 for diagnosing depression [27].

Clinical Global Impression Scale-Severity (CGI-S)

The CGI, which is widely used in psychiatry, consists of a three-item observer-rated scale. It assesses the severity of illness (CGI-S), global improvement or change in symptoms (CGI-I), and therapeutic response (or efficacy) [22]. The CGI-S employs a seven-point scale to gauge the severity of illness based on responses to various questions, ranging from (one) normal and not at all ill to (seven) extremely ill.

Statistics analyses

Analyses were performed using the IBM SPSS Statistics for Windows, Version 25 (Released 2017; IBM Corp., Armonk, New York, United States). Categorical variables were presented as frequencies and percentages, while continuous variables were expressed as mean ± standard deviation, or median (interquartile range). The normal distribution of numerical variables was assessed by visual (histograms and probability plots) and analytical (Shapiro-Wilk) methods. The student t-test was used to assess the difference between the two groups for normally distributed numerical data. For non-normally distributed data, the Mann-Whitney U test was used to assess the difference in rank distribution between the two groups. Comparison of categorical variables was performed using the chi-square test (Pearson chi-square) or Fisher's exact test. The Pearson correlation test was used to assess the relationship between variables that had a normal distribution, while the Spearman correlation analysis was used for those that had a non-normal distribution. All analyses were considered statistically significant at p 0.05.

## Results

The sample consisted of two groups: HC (n=19) and MDD (n=24), matched for age, sex, and BMI. The mean age was 12.5±3.1 years for the HC group and 12.8±3.2 years for the MDD group. The CDI and CGI-S scores were significantly higher in the MDD group than in the HC group (all, p<0.001). The demographic and clinical characteristics of the subjects are presented in Table 1.

**Table 1:** Demographic and clinical characteristics of study subjects BMI: body mass index; CDI: Child Depression Inventory; CGI-S: Clinical Global Impression Scale-Severity; HC: healthy control; kg: kilogram; MDD: major depressive disorder; m: meter ^a^Student t test; ^b^Chi-square test; ^c^Fisher exact test; p<0.05, statistically significant

Characteristics		MDD (n=24)	HC (n=19)	p-value
Age (years)	mean±sd	12.8±3.2	12.5±3.1	0.783^a^
Gender				
Female	n (%)	15 (62.5)	13 (68.4)	0.934^b^
Male	n (%)	9 (37.5)	6 (31.6)	
Family history of psychiatric disorder	n (%)	9 (37.5)	1 (5.3)	0.026^ c^
BMI (kg/m^2^)	mean±sd	19.6±3.0	19.8±2.9	0.845^a^
CDI	mean±sd	35.1±5.0	4.9±2.8	<0.001^a^
CGI-S	mean±sd	5.3±0.8	1.0±0.0	<0.001^a^

The TDH parameters and IMA levels did not show statistically significant differences between the two groups, as shown in Table 2. Although not statistically significant, the levels of native thiol and total thiol were found to be higher in the case group (all, p>0.05). 

**Table 2:** Comparison of oxidative stress parameters between the two groups ABSU: absorbance unit; HC: healthy control; IMA: ischemia-modified albumin; MDD: major depressive disorder ^a^Student t test; ^b^Mann-Whitney U test; p<0.05, statistically significant

Parameters		MDD (n=24)	HC (n=19)	p-value
Native thiol	mean±sd	457.1±53.2	427.1±91.5	0.215^a^
Total thiol	mean±sd	502.6±54.6	471.3±90.1	0.194^a^
Disulfide	n (%)	22.9 (5.4)	22.9 (6.0)	0.441^b^
(Disulfide/native thiol)*100	n (%)	5.3 (1.6)	4.9 (2.6)	0.826^b^
(Disulfide/total thiol)*100	n (%)	4.8 (1.3)	4.5 (2.1)	0.826^b^
(Native thiol/total thiol)*100	n (%)	90.4 (2.7)	90.9 (4.2)	0.826^b^
IMA (ABSU)	mean±sd	0.6±0.3	0.7±0.3	0.494^a^

Participants' CGI-S and CDI scores had a significantly high positive correlation (p<0.001). Scale scores and oxidative stress parameters were not significantly correlated (Table 3).

**Table 3:** Correlating of scale scores with oxidative stress parameters CDI: Child Depression Inventory; CGI-S: Clinical Global Impression Scale–Severity; IMA: ischemia-modified albumin ^a^Pearson correlation; ^b^Spearman correlation; p<0.05, statistically significant

Variables		CDI	CGI-S
CGI-S	r	0.835	-
	p^a^	<0.001	-
Native thiol	r	0.243	0.174
	p^a^	0.117	0.266
Total thiol	r	0.250	0.183
	p^a^	0.106	0.241
Disulfide	rho	0.084	0.075
	p^b^	0.592	0.631
IMA	r	-0.183	-0.160
	p^a^	0.240	0.304

## Discussion

This study compared MDD and HC groups to analyze possible associations between childhood MDD and oxidative stress biomarkers (TDH and IMA). We did not observe any significant differences between the two groups, but there was a noticeable trend towards increased antioxidants in those with MDD. In particular, the higher levels of native thiol and total thiol in the MDD group highlight the complex nature of these biological markers. Another important finding of our study is that there was no correlation between disease severity and the levels of TDH and IMA. To the best of our knowledge, these findings provide new insights into childhood MDD.

In contrast to our study, previous studies have reported significant associations between oxidative stress and depressive disorders [8,9,28]. However, we did not find such a difference in the group of children. Our small sample size may have influenced this. The lack of increased oxidative stress may also be explained by the fact that the subjects were children, did not have high BMI, and had no additional comorbidities. Advancing age, high BMI [29], and comorbid conditions [30,31] contribute to the increase in oxidative stress. Despite this, we observed a slight upward trend in antioxidant markers in children with MDD. These findings may suggest that the increase in oxidative stress is a process that progresses with age, possibly due to the cumulative effects of reactive oxygen species over time, decreased efficiency of antioxidant defenses, and the onset of age-related comorbidities such as higher BMI and chronic diseases.

The literature on the relationship between pediatric MDD and oxidative stress is rather limited. Case-control studies investigating this association have examined various biomarkers such as total oxidant status (TOS), total antioxidant status (TAS), oxidative stress index (OSI), malondialdehyde (MDA), superoxide dismutase (SOD), serum S100B, 8-isoP-U, and lipoperoxide levels. It has been reported that oxidative stress markers are elevated in patients with MDD [15,17,32]. One study investigating oxidative stress through a comparative analysis of serotonin (5-HT) and serum uric acid levels between adolescents diagnosed with depression and HC suggests a possible association between adolescent depression and oxidative damage to neuronal DNA in the brain [16]. Although methodologically similar, the parameters used to assess oxidative stress vary, resulting in inconsistent findings. The absence of the oxidative stress parameters we used in our study has also made it difficult to compare results directly.

A case-control study by Ozturk et al. (2023) reported that TDH was impaired in adolescents aged 13-18 years diagnosed with MDD compared with HCs, suggesting that it may contribute to the etiopathogenesis of MDD. In that study, lower levels of native thiol were observed in adolescents diagnosed with MDD [18]. The presence of comorbid mental disorders in more than half of the adolescents and the exclusive focus on the adolescent age group may have contributed to the observed increase in oxidative stress. In our study, patients with MDD did not have comorbid mental disorders, and the age range of the participants was between seven and 17 years. The effect of the aging process on age-related changes is modest in young life but increases rapidly with age due to the nature of aging [33]. Thus, oxidative stress may be increased during the transition from childhood to adolescence. In this research, the tendency for higher native and total thiol levels in the MDD group may be related to activation of the antioxidant system, especially depending on the age group.

Although, similar to our study, TAS, which is another antioxidant parameter, was found to be lower in children aged seven to 17 years with a first diagnosis of MDD in whom comorbid psychiatric disorders had been excluded [17]. This suggests that there may be a link between oxidative stress and the stage of development of the antioxidant system.

In this study, we found no association between disease severity and levels of TDH and IMA. A study assessing antioxidant capacity in adolescent patients with depression by glutathione (GSH) analysis using MRI spectroscopy showed significantly lower GSH levels in patients but without any association with disease severity [34]. A case-control study similar to ours, which examined various oxidative parameters in adolescents diagnosed with first-episode MDD, found no association between oxidative stress parameters and disease severity [32]. Interestingly, a recent study found that an increase in TDH impairment correlated with depression scores, suggesting that this condition may be proportional to disease severity [18].

Strengths and limitations

Strengths of the study include its exclusive examination of medication-naive children with MDD, thereby minimizing confounding variables associated with prior treatment. The lack of comorbid mental disorders in our study population suggests that the observed association between oxidative stress and MDD severity may be more directly attributable to the effects of MDD itself rather than to confounding factors related to other psychiatric conditions. Furthermore, the inclusion of subjects with a normal BMI increases the internal validity of the study. In addition, the absence of chronic diseases in both groups further strengthens the comparison between cases and controls.

Limitations of our study include the limited sample size, the wide age range of children, the lack of measurement of 12-hour overnight fasting, which was self-reported by the family, and the sole reliance on venous blood sampling to assess oxidative stress. We call for future studies to include larger and more diverse cohorts, possibly incorporating longitudinal approaches, to validate and extend the current findings.

## Conclusions

In conclusion, our study sheds light on the complex relationship between oxidative stress and MDD in a pediatric population. The observed increase in native and total thiol levels in the MDD group suggests possible implications for the antioxidant balance in the pathophysiology of MDD. Our study did not find a direct association between oxidative stress parameters and disease severity in children with MDD. The findings highlight the need for further investigation of oxidative stress mechanisms in MDD. Future studies examining different subgroups and using a broader range of biomarkers are essential for a comprehensive understanding of the complex interplay between oxidative stress and pediatric MDD.
